# Lemierre's Syndrome Due to *Klebsiella pneumoniae* Results in Pulmonary Abscess Complications in a Patient With Diabetes: A Rare Case Report

**DOI:** 10.1155/crdi/8176530

**Published:** 2024-12-23

**Authors:** Trung Dinh Ngo, Cuong Thai Nguyen, Nam Ho

**Affiliations:** Surgical and Transplant Intensive Care Unit, 108 Military Central Hospital, Hanoi, Vietnam

## Abstract

**Background:** Lemierre's syndrome (LS), first described by Andre Lemierre in the early 20th century, is a rare but potentially life-threatening condition typically caused by *Fusobacterium necrophorum*. However, recent literature has reported cases of LS caused by various other bacteria, including *Klebsiella pneumoniae*. In this report, we present a rare case of LS in a patient with diabetes caused by *K. pneumoniae*.

**Case Report:** A 62-year-old Vietnamese male with a history of type 2 diabetes mellitus, presented with an 8-day history of progressive left neck swelling, fever, dysphagia, odynophagia, and reduced appetite. Despite initial antibiotic therapy, his condition deteriorated, leading to pulmonary abscesses and septic shock. Abscess content culture revealed K. pneumoniae. The patient required intubation, mechanical ventilation, and surgical drainage of the neck abscess. Treatment with meropenem, along with glycemic control, led to clinical improvement. The patient was subsequently extubated, achieved complete wound healing, and was discharged with normal biochemical parameters.

**Conclusion:** This case highlights that LS can be caused by pathogens not initially outlined by Andre Lemierre, such as *K. pneumoniae*. Clinicians should consider a broader spectrum of causative organisms when there is a strong clinical suspicion of LS and adjust antimicrobial coverage accordingly. The association between *K. pneumoniae*-related LS and diabetes mellitus warrants further investigation, as current evidence suggests that diabetes may predispose patients to this particular pathogen.

## 1. Background

Lemierre's syndrome (LS) is rare and potentially life-threatening condition, with an incidence of 1 case per million annually [1]. It is characterized by a severe infection originating in the head and neck region that progresses to form thrombophlebitis of the internal jugular vein [2]. This condition was first described by French physician Andre Lemierre in the early 20th century with 20 cases of anaerobic septicemia, of which 18 resulted in death. Classically, LS is associated with infections caused by the bacterium *Fusobacterium necrophorum*, which accounts for 81.7% of the cases [3, 4]. However, recent literature has documented emerging cases of LS caused by various other bacteria, including *Klebsiella pneumoniae* [5, 6].

*K. pneumoniae* is a Gram-negative bacterium known for causing a range of infections, particularly in immunocompromised individuals or those with underlying health conditions [7]. LS caused by *K. pneumoniae* is an atypical presentation that requires further investigation that warrants further investigation. Its clinical features, management strategies, and outcomes may differ from the classical form of the syndrome caused by *Fusobacterium necrophorum*. Understanding the unique characteristics and challenges posed by this variant of LS is crucial for prompt diagnosis and appropriate therapeutic interventions.

In this report, we present a rare case of LS caused by *K. pneumoniae* in a patient with diabetes and review the relevant literature.

## 2. Case Report

### 2.1. History of Present Illness

A 62-year-old Vietnamese male, with a history of poorly controlled type 2 diabetes mellitus, presented with an 8-day history of progressive left neck swelling and fever. He denied any prior dyspnea, upper respiratory tract infection, dental problems, or procedures. He was admitted to the local hospital with a diagnosis of left neck lymphadenitis. A lymph node biopsy revealed chronic lymphadenitis. Despite initial treatment with amoxicillin (2 g per day) and doxycycline (200 mg per day), his condition deteriorated, with increasing neck swelling, pain, dysphagia, odynophagia, and reduced appetite.

### 2.2. Physical Examination

**Figure 1:**
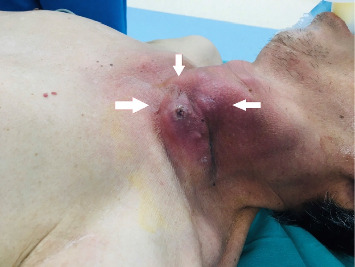
A left neck swelling was 10 × 12 cm in size with cardinal features of acute inflammation (rubor, calor, tumor, dolor, and functio laesa).

On admission examination, the patient was febrile (39°C), conscious, dyspneic with a respiratory rate of 28–30 cycles per minute, and a saturation of peripheral oxygen (SPO_2_) of 85%. Pulmonary auscultation showed respiratory crackles on both sides. In additionally, he was hypotensive with a blood pressure of 80/50 mmHg (on noradrenaline 0.1 μg/kg/min). A left neck swelling (10 × 12 cm) with cardinal features of acute inflammation (rubor, calor, tumor, dolor, and functio laesa) was noted (Figure 1). No further abnormality was found upon the examination of the nervous system, digestive system, kidneys.

Laboratory examinations: laboratory investigations revealed leukocytosis (12 × 10^3^/μL), neutrophilia (86% of total blood leukocytes), anemia (3.76 × 10^6^ red bleed cells/μL, hemoglobin level 10.6 g/dL, and hematocrit level 24.4%), and thrombopenia (125 × 10^3^ platelets/μL). Furthermore, an elevated procalcitonin (16.3 ng/mL) and hyperglycemia (glucose level: 20 mmol/L) were found. An arterial blood gas test revealed metabolic acidosis with a pH 7.3, PaCO2 32 mmHg, PaO2 level of 83 mmHg (the fraction of inspired oxygen (FiO2) of 40%), and a bicarbonate level of 18 mEq/L, lactat 4 mmol/l. A conclusive SOFA score of 6 was documented.

### 2.3. Imaging Examinations

**Figure 2:**
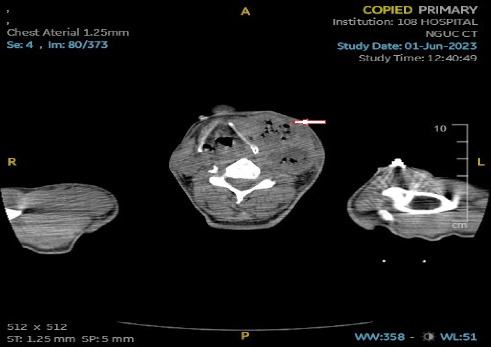
Computed tomography of the left and anterior neck region shows a mass with gas-fluid levels, border is not clear, size 40 × 56 × 106 mm.

**Figure 3:**
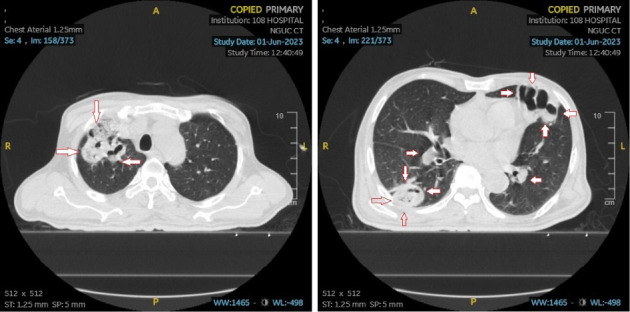
Computed tomography image of the chest shows multiple abscesses in both lungs, the largest abscess size of the right lung is 48 × 50 mm, the largest abscess size of the left lung is 36 × 54 mm.

**Figure 4:**
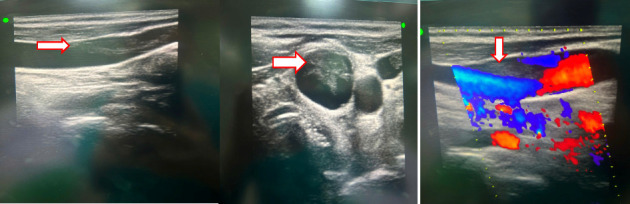
The ultrasound image shows left internal jugular vein thrombosis.

An ultrasound of the neck area showed an irregular echogenic mass measuring 60 × 33 mm, with fluid present within the mass. A computed tomography (CT) image of the left neck region (Figure 2) demonstrated a multiloculated abscess with gas-fluid levels (arrowheads) and an unclear border, measuring 40 × 56 × 106 mm. The CT also revealed a mass effect on the oropharynx, hypopharynx and larynx. However, no thrombosis of the internal jugular vein (IJV) was observed (Figure 2). A CT scan of the chest showed peribronchial consolidation and ground-glass opacities with cavitary nodules in both lungs, as well as multiple abscesses. The largest abscess in the right lung measured 48 × 50 mm, while the largest abscess in the left lung was 36 × 54 mm. There was no evidence of pulmonary thromboembolism (Figure 3). However, the ultrasound image shows left internal jugular vein thrombosis (Figure 4).

### 2.4. Initial Diagnosis and Management

The patient was diagnosed with septic shock secondary to a left neck abscess with pulmonary abscess complications. An hour-1 Surviving Sepsis Campaign bundle [8] of care was promptly initiated. This included an empiric antibiotic therapy with meropenem and vancomycin. Specifically, a regimen of meropenem 3 g per day and vancomycin 2 g per day was initially intravenously administrated. The dose of vancomycin for subsequent days was adjusted according to the blood concentration of vancomycin, with a target of concentration of vancomycin 20–25 mcg/mL. Furthermore, appropriate fluid resuscitation (sodium chloride 0.9% and serum lactate level guided) and vasopressor support (noradrenaline) measures were employed. Moreover, a glycemic control with continuous intravenous rapid insulin was used with a blood glucose target level of 8–10 mmol/L. At the same time, the patient was intubated, mechanically ventilated, and underwent surgical drainage of the neck abscess.

### 2.5. Microbiological Identification of the Causative Agent

As a part of the bundle, samples of blood, sputum, and purulent abscess were also collected for culture. Abscess culture revealed *K. pneumoniae*. However, blood culture results were repeatedly negative. Other microbiological tests were also negative. These include polymerase chain reaction (PCR) for bacteria identification of blood, Ziehl–Neelsen-stained acid-fast bacillus test (AFB) and PCR for *Mycobacterium tuberculosis* of sputum sample.

### 2.6. Definite Diagnosis

LS caused by K. pneumoniae, complicated by lung abscesses and septic shock.

### 2.7. Clinical Course and Outcome

**Figure 5:**
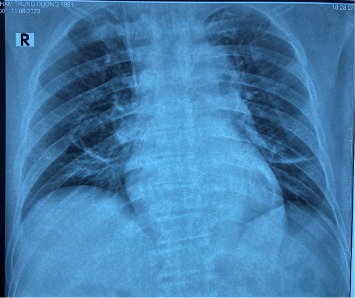
Chest x-ray on the 4th day after admission shows clear lungs.

With continued antibiotic therapy, glycemic control, and supportive care, the patient's condition improved. On the 4th day of admission, he was awake and his respiratory status had improved. He was afebrile, and an arterial blood gas test revealed a pH of 7.49, PaCO2 of 38 mmHg, PaO2 of 156 mmHg (FiO2 40%), and a bicarbonate level of 28 mEq/L. A chest x-ray demonstrated clear lungs (Figure 5). Subsequently, the patient was extubated. By the 14^th^ day of admission, he was clinically stable. Laboratory tests showed a leukocyte count of 6 × 10^3^/μL, with70% neutrophils, and a procalcitonin level of 0.5 ng/mL. Normoglycemia was achieved before his discharge from the hospital. The patient was discharged on the 15^th^ day of admission, following complete wound healing and the normalization of his biochemical parameters.

## 3. Discussion

The case report presents an uncommon complication of oropharyngeal infection in a middle-aged man with a medical history of poorly controlled type 2 diabetes mellitus. Before the advent of antibiotics, LS had its highest reported occurrence [9]. Following the extensive utilization of penicillin during the 1960s and 1970s, the occurrence of this syndrome significantly decreased, and LS became known as the “forgotten disease” [10]. However, in the 1970s, instances of LS started reappearing, partially attributed to enhanced diagnostic imaging techniques [11]. Currently, the syndrome remains rare, with a global estimated incidence of 1 case per 1 million people [1]. LS usually affects previously healthy young individuals, specifically adolescents and young adults, with a median age range of 19–22 years [9]. In a recent review by Riordan, 89% of the 222 examined LS cases were diagnosed in individuals aged 10–35 years [12].

*Fusobacterium necrophorum* and *Fusobacterium nucleatum* are the predominant culprits in LS cases [13]. These bacteria can cause invasive disease secondary to multiple virulence factors, including endotoxins and exotoxins [9]. However, additional microorganisms, such as Streptococcus species, Bacteroides species, *Staphylococcus aureus*, and *Klebsiella pneumoniae*, have been documented in separate case studies [14, 15]. The participation of *K. pneumoniae* in the development of LS is infrequent, with only a few documented cases in the past, all involving individuals with diabetes [6, 16–18]. A recent review found that approximately 50% of diabetic patients with deep neck abscesses had *K. pneumoniae* isolated from the abscess collections [19]. Huang et al. [20] found that 98.4% of their diabetic patients had infections caused by *K. pneumoniae*. This raises the question of whether diabetes mellitus plays a role in the predisposition to this organism or if it is merely a matter of chance.

Patients with type 2 diabetes mellitus face a heightened susceptibility to infections due to decreased neutrophil activity [21]. This increased susceptibility to *K. pneumoniae* among individuals with type 2 diabetes mellitus can be attributed to certain mechanisms. Specifically, the hypermucoviscosity phenotype of *K. pneumoniae*, particularly the K1/K2 strains, exhibits resistance to phagocytosis [22]. Glycemic uncontrol significantly diminishes the ability to phagocytose virulent K1/K2 *K. pneumoniae*. Furthermore, Lin et al. [23] demonstrated that elderly patients with poorly managed blood sugar levels experience reduced phagocytic activity.

The presentation and clinical progression of LS can be divided into 3 main phases: (1) oropharyngeal infection with subsequent febrile episodes and rigors 4–7 days after the initial illness; (2) infection extension to the parapharyngeal space of the neck with thrombophlebitis of the internal jugular vein phases; and (3) septic emboli phases [24]. The lungs are the most frequently affected organ (85%), but other organs, including joints, liver, kidney, brain, bones, heart, and meninges, can all be involved. Bacteremia is associated with fever, lethargy, or shock, as well as end-organ damage. Septic shock occurs in approximately 7% of the cases, whereas acute respiratory distress syndrome requiring mechanical ventilation may affect up to 10% of the patients [9].

The primary treatment approach for LS involves antibiotic therapy [25], typically starting with a beta-lactamase-resistant beta-lactam antibiotic due to reported cases of penicillin failure, often due to *F. necrophorum* producing beta-lactamase. When culture results and susceptibility data are accessible, antibiotics should be customized accordingly [9]. Alternative options include carbapenem, clindamycin, or metronidazole. Antibiotic therapy is continued for 6 weeks in most patients to achieve appropriate penetration into fibrin clots [26].

Surgical intervention may be required when abscesses form, respiratory distress arises from pulmonary thrombosis, metastasis occurs, or when thrombus extends into critical areas like the mediastinum or cerebrum. Incising and draining the abscess at affected sites may be recommended to control infection [9].

Anticoagulation therapy in LS is controversial due to the absence of controlled trials. Some authors have noted clinical pictures of persisting fever, worsening clinical condition, and ongoing radiographic evidence of continued pulmonary emboli until comprehensive anticoagulant therapy is initiated [27]. In uncomplicated LS lacking extensive clot burden, resolution often occurs with appropriate antibiotic treatment and supportive care, typically obviating the need for anticoagulation. Although there are no controlled trials affirming this approach, anticoagulation is commonly advised when the thrombus extends into cerebral sinuses, involves large or bilateral clot burdens, or when a patient does not show improvement within the initial 72 h despite appropriate antibiotic and/or surgical interventions [9].

## 4. Conclusion

LS is a rare condition that can be caused by pathogens not initially outlined by Andre Lemierre. While *Fusobacterium necrophorum* remains the most common causative agent, clinicians should be aware of the possibility of other organisms, such as *K. pneumoniae*, particularly when there is a strong clinical suspicion. In such cases, broader antimicrobial coverage should be considered.

In LS cases associated with K. pneumoniae, it is noteworthy that the affected patients had a history of diabetes mellitus. The current evidence suggests that diabetes mellitus may play a role in predisposing patients to infections caused by this pathogen. The increased susceptibility to *K. pneumoniae* among diabetic patients may be attributed to impaired neutrophil function and reduced phagocytic activity, especially in the presence of poor glycemic control. Therefore, clinicians should maintain a high index of suspicion for atypical causative agents in LS, particularly in patients with underlying conditions such as diabetes mellitus. Prompt recognition, appropriate antimicrobial therapy, and management of comorbidities are crucial for improving patient outcomes in these rare but potentially life-threatening cases.

## Data Availability

All data related to this case report are included within the manuscript. Additional clinical details can be made available by the corresponding author upon reasonable request, ensuring compliance with patient confidentiality regulations.
